# Supplementary material for: Accumulation of health complaints is associated with persistent musculoskeletal pain two years later in adolescents: The Fit Futures study
Source: PLoS One. 2022 Dec 29;17(12):e0278906. doi: 10.1371/journal.pone.0278906 (PMC9799295; doi:10.1371/journal.pone.0278906)
Supplement: S1 Table — (DOCX) [file pone.0278906.s001.docx]

**Table S1: Comparison of baseline characteristics of respondents and non-respondents ^a^ of the follow-up study (FF2)**

| **Characteristics** | **Respondents**  **(n = 551)** | **Non-respondents ^a^ (n=238)** | **P-value** |
| --- | --- | --- | --- |
| Age, y, median (min-max) | 16 (15-19) | 16 (15-19) | P = 0.49 |
| Sex, girls, n (%) | 286 (51.9) | 78 (32.8) | P < 0.01 |
| Parents` employment status, n (%)  Both parents are employed  One / neither parent is currently employed  Don’t know  *Missing, n* | 415 (75.7)  113 (20.6)  20 (3.6)  *3* | 163 (68.5) 64 (26.9)  11 (4.6)  *0* | P = 0.11 |
| Parents` education, n (%)  At least one parent with higher education  Primary / secondary school  Don’t know  *Missing, n* | 281 (52.2) 126 (23.4)  131 (24.3)  *13* | 95 (41.5)  56 (24.5)  78 (34.1)  *9* | P=0.01 |
| Asthma, n (%)  Yes  No or don`t know  *Missing, n* | 63 (11.6)  482 (88.4)  *6* | 34 (14.3) 203 (85.7)  *1* | P=0.28 |
| Atopic eczema, n (%)  Yes  No or don`t know  *Missing, n* | 73 (13.3) 474 (86.7) *4* | 31 (13.1)  206 (86.9)  *1* | P=0.92 |
| Allergic rhinitis, n (%)  Yes  No or don`t know  *Missing, n* | 54 (9.9)  490 (90.1) *7* | 20 (8.4)  217 (91.6)  *1* | P=0.51 |
| Headache, yes ^b^, n (%)  *Missing, n* | 185 (33.6) 0 | 78 (32.9)  *1* | P=0.86 |
| Abdominal pain, yes ^c^, n (%)  *Missing, n* | 85 (15.5) *1* | 36 (15.3)  *2* | P=0.94 |
| Psychological distress ^d^, median (min-max)  Psychological distress (cat.) ^d^, n (%)  > 1.85  ≤ 1.85  *Missing, n* | 1.3 (1 - 3.5)  80 (14.7) 466 (85.3)  *5* | 1.2 (1-3.6)  33 (14.3)  197 (85.7)  *8* | P=0.07  P=0.91 |
| Accumulated number of health complaints ^e^, median (min-max)  *Missing, n* | 1 (0-5) 19 | 1 (0-5)  15 | P=0.83 |
| ^a^ Non-respondents include both those who did not attend the follow-up study at all, and those who did not answer the pain outcome of interest, ^b^ Headache minimum 1-6 days a month; ^c^ Abdominal pain minimum once a week the last two months; ^d^ Psychological distress, Hopkins Symptom Checklist-10 (HSCL-10), continuous (1-4), categorized (cut-off >1.85 indicates the presence of psychological distress); ^e^ The variable includes asthma, atopic eczema, allergic rhinitis, headache, abdominal pain, psychological distress and other reported health complaints (diabetes type 1, ADHD, psoriasis, arthritis, anaemia, sleep disorder, food allergy or intolerance) | | | |
