# Supplementary material for: Accumulation of health complaints is associated with persistent musculoskeletal pain two years later in adolescents: The Fit Futures study
Source: PLoS One. 2022 Dec 29;17(12):e0278906. doi: 10.1371/journal.pone.0278906 (PMC9799295; doi:10.1371/journal.pone.0278906)
Supplement: S2 Table — (DOCX) [file pone.0278906.s002.docx]

**Table S2: Logistic regression analyses of the associations between health complaints (asthma, atopic eczema, allergic rhinitis) at baseline and persistent musculoskeletal
pain ^a^ at follow-up, excluding participants who responded “don`t know” to the baseline health complaints questions**

| **Health complaints** |  |  |  |  |  |
| --- | --- | --- | --- | --- | --- |
|  | **Exposed cases ^c^** | **Crude**  **OR (95% CI)** | ***P*-value** | **Adjusted ^d^**  **OR (95% CI)** | ***P*-value** |
| **Asthma ^b^** | 12 | 1.66 (0.83, 3.30) | 0.15 | 1.76 (0.87, 3.55) | 0.12 |
| **Atopic eczema ^b^** | 12 | 1.36 (0.68, 2.70) | 0.38 | 1.12 (0.54, 2.33) | 0.76 |
| **Allergic rhinitis ^b^** | 10 | 1.47 (0.70, 3.08) | 0.31 | 1.31 (0.61, 2.79) | 0.49 |
| Abbreviations: Odds ratio (OR), Confidence Interval (CI). N= 451 - 507 in adjusted models  ^a^ Persistent musculoskeletal pain is defined as weekly musculoskeletal pain for three months or more  ^b^ The reference is “no” ^c^ Number of participants with the specific health complaint at baseline *and* persistent musculoskeletal pain at follow-up  ^d^ Adjusted for sex, parents` employment status and other health complaints (yes/no) | | | | | |
